# Supplementary material for: Incorporating functional priors improves polygenic prediction accuracy in UK Biobank and 23andMe data sets
Source: Nat Commun. 2021 Oct 18;12:6052. doi: 10.1038/s41467-021-25171-9 (PMC8523709; doi:10.1038/s41467-021-25171-9)
Supplement: Supplementary file 1 — Supplementary Information [file 41467_2021_25171_MOESM1_ESM.pdf]

Supplementary Information

Supplementary Figures

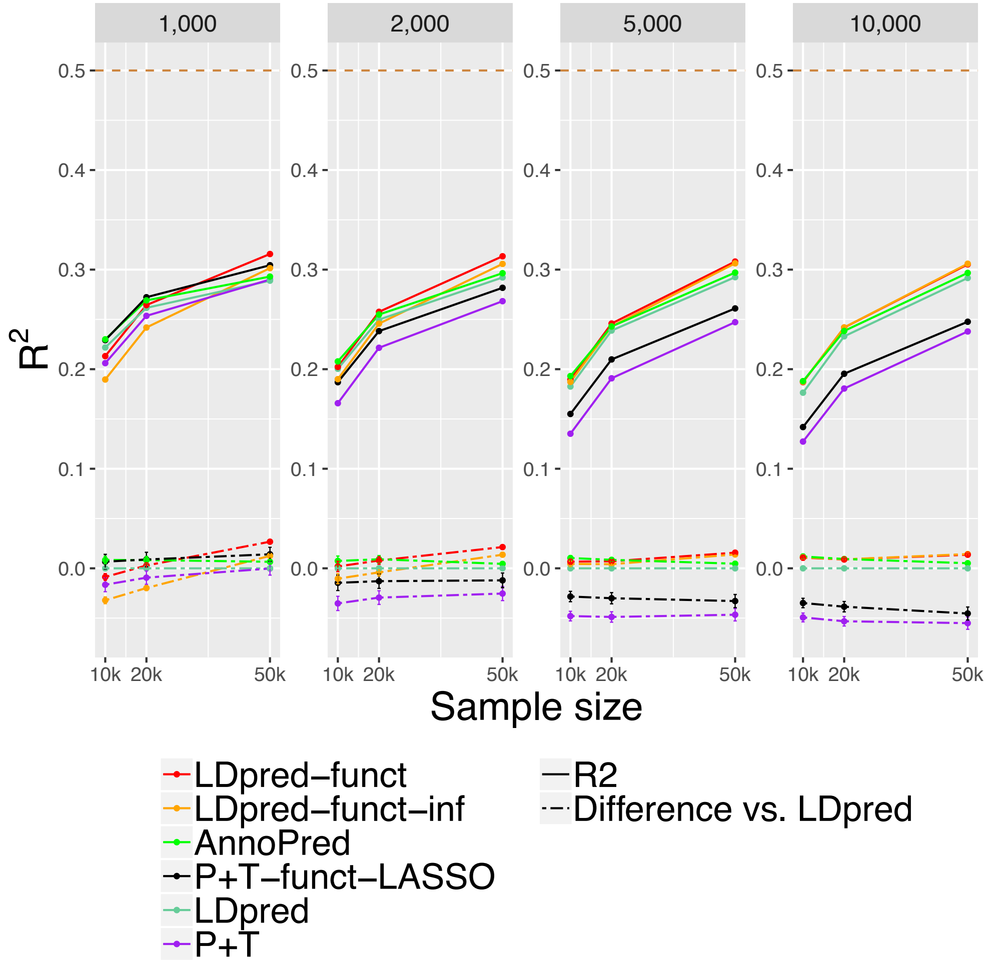

Supplementary Figure 1: Accuracy of 6 polygenic prediction methods in simulations using UK Biobank genotypes, for 4 values of the number of causal variants. We report results for P+T, LDpred, P+T-funct-LASSO, AnnoPred, LDpred-funct-inf and LDpred-funct in chromosome 1 simulations with 1,000 causal variants (very sparse architecture), 2,000 causal variants (sparse architecture), 5,000 causal variants (polygenic architecture) and 10,000 causal variants (very polygenic architecture). Results obtained in a validation set with n=7,585 are averaged across independent 100 simulations. Top dashed line denotes simulated SNP-heritability of 0.5. Bottom dashed lines denote differences vs. LDpred; error bars represent 95% confidence intervals. Numerical results are reported in Supplementary Table 1 and Supplementary Table 2. Source data are provided as a Source Data file.

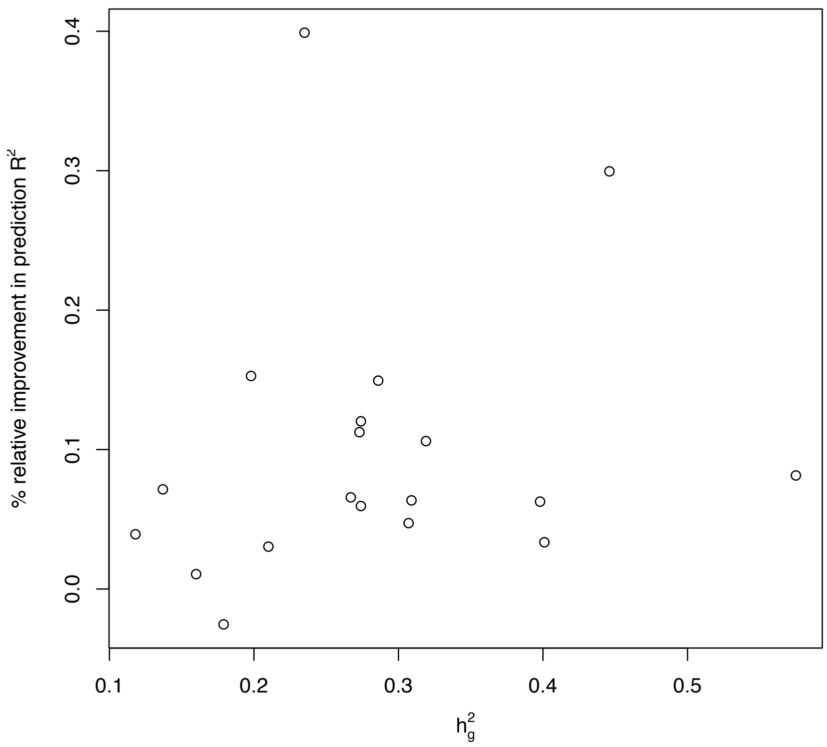

Supplementary Figure 2: Relative improvement of LDpred-funct vs. LDpred as a function of h^2_g. We plot relative improvement vs. h^2_g (measured on the observed scale for binary traits) for 19 UK Biobank traits; we excluded two sex-specific traits, age at menarche and balding type I. We observed a correlation of 0.186 across the 19 traits, which was non-significant ($P = 0.4$ from two-sided t-test). Source data are provided as a Source Data file.

Supplementary Tables

# Causal variants	Model	Training sample size 10,000 Average $R^2(s.e.)$	20,000 Average $R^2(s.e.)$	50,000 Average $R^2(s.e.)$
1,000	P+T	0.2061 (0.0022)	0.2536 (0.0021)	0.2900 (0.0019)
	LDpred	0.2218 (0.0024)	0.2616 (0.0021)	0.2889 (0.0018)
	P+T-funct-LASSO	0.2292 (0.0024)	0.2723 (0.0024)	0.3044 (0.002)
	AnnoPred	0.2300 (0.0025)	0.2691 (0.0027)	0.2930 (0.0019)
	LDpred-funct-inf	0.1896 (0.0018)	0.2419 (0.0019)	0.3015 (0.0019)
	LDpred-funct	0.2131 (0.002)	0.2644 (0.0021)	0.3157 (0.002)
2,000	P+T	0.1658 (0.0022)	0.2215 (0.0026)	0.2683 (0.0029)
	LDpred	0.2004 (0.0028)	0.2498 (0.0023)	0.2921 (0.0015)
	P+T-funct-LASSO	0.1869 (0.0026)	0.2383 (0.0028)	0.2817 (0.0031)
	AnnoPred	0.2078 (0.0018)	0.2549 (0.0028)	0.2964 (0.0016)
	LDpred-funct-inf	0.1900 (0.0015)	0.2458 (0.0015)	0.3057 (0.0016)
	LDpred-funct	0.2023 (0.0016)	0.2576 (0.0016)	0.3134 (0.0017)
5,000	P+T	0.1352 (0.0016)	0.1909 (0.002)	0.2472 (0.0024)
	LDpred	0.1826 (0.0017)	0.2388 (0.0013)	0.2924 (0.0013)
	P+T-funct-LASSO	0.1550 (0.0018)	0.2098 (0.0021)	0.2610 (0.0026)
	AnnoPred	0.1931 (0.0013)	0.2429 (0.0021)	0.2970 (0.0014)
	LDpred-funct-inf	0.1872 (0.0012)	0.2430 (0.0013)	0.3063 (0.0014)
	LDpred-funct	0.1895 (0.0012)	0.2458 (0.0013)	0.3081 (0.0014)
10,000	P+T	0.1273 (0.0015)	0.1806 (0.002)	0.2379 (0.0024)
	LDpred	0.1764 (0.0016)	0.2330 (0.0012)	0.2916 (0.0012)
	P+T-funct-LASSO	0.1419 (0.0017)	0.1954 (0.0022)	0.2477 (0.0026)
	AnnoPred	0.1880 (0.0013)	0.2384 (0.0021)	0.2967 (0.0013)
	LDpred-funct-inf	0.1873 (0.0012)	0.2419 (0.0012)	0.3059 (0.0013)
	LDpred-funct	0.1870 (0.0013)	0.2418 (0.0012)	0.3053 (0.0012)

Supplementary Table 1: Accuracy of 6 polygenic prediction methods in simulations using UK Biobank genotypes, for 4 values of the number of causal variants. We report results for P+T, LDpred, P+T-funct-LASSO, AnnoPred, LDpred-funct-inf and LDpred-funct in chromosome 1 simulations with 1,000 causal variants (very sparse architecture), 2,000 causal variants (sparse architecture), 5,000 causal variants (polygenic architecture) and 10,000 causal variants (very polygenic architecture). Results are averaged across 100 simulations. We report standard errors in parentheses. For SBayesR simulations at N=10K, we obtained average prediction R^2 of 0.2754 (0.002), 0.2381 (0.001), 0.1983 (0.001) and 0.1827 (0.001) for 1,000, 2,000, 5,000 and 10,000 causal variants respectively. However, for all SBayesR simulations at N=20K and N=50K, we obtained average prediction R^2 of 0.0005 (0.0001) and $h_g^2 = 0.99$ in 100/100 simulations, perhaps because the algorithm failed to converge.

(a)

# Causal variants	Model	Training sample size: 10,000 Diff. $R^2 (s.e.)$	20,000 Diff. $R^2 (s.e.)$	50,000 Diff. $R^2 (s.e.)$
1,000	P+T	0.0069 (0.0018)	0.0106 (0.0016)	0.0254 (0.0015)
	P+T-funct-LASSO	-0.0162 (0.002)	-0.0081 (0.0018)	0.011 (0.0016)
	LDpred	-0.0087 (0.0017)	0.0028 (0.0013)	0.0267 (8e-04)
	AnnoPred	-0.0169 (0.0018)	-0.0054 (0.0017)	0.0203 (0.0012)
	LDpred-funct-inf	0.0235 (8e-04)	0.0225 (6e-04)	0.0142 (6e-04)
	LDpred-funct	0	0	0
2,000	P+T	0.0365 (0.0019)	0.0361 (0.0022)	0.0451 (0.0026)
	P+T-funct-LASSO	0.0153 (0.0023)	0.0194 (0.0024)	0.0317 (0.0027)
	LDpred	0.0019 (0.0026)	0.0078 (0.0019)	0.0213 (7e-04)
	AnnoPred	-0.0056 (0.0012)	-0.0012 (0.0012)	0.0167 (7e-04)
	LDpred-funct-inf	0.0123 (5e-04)	0.0118 (5e-04)	0.0077 (4e-04)
	LDpred-funct	0	0	0
5,000	P+T	0.0544 (0.0016)	0.055 (0.0018)	0.0609 (0.0021)
	P+T-funct-LASSO	0.0345 (0.0017)	0.036 (0.0019)	0.0471 (0.0023)
	LDpred	0.0067 (0.0013)	0.007 (7e-04)	0.0157 (5e-04)
	AnnoPred	-0.0035 (5e-04)	-0.0015 (6e-04)	0.011 (6e-04)
	LDpred-funct-inf	0.0023 (3e-04)	0.0029 (3e-04)	0.0018 (2e-04)
	LDpred-funct	0	0	0
10,000	P+T	0.0597 (0.0016)	0.0612 (0.002)	0.0674 (0.0024)
	P+T-funct-LASSO	0.0451 (0.0017)	0.0464 (0.0022)	0.0576 (0.0026)
	LDpred	0.0107 (0.0013)	0.0089 (5e-04)	0.0136 (5e-04)
	AnnoPred	-0.0011 (3e-04)	-4e-04 (5e-04)	0.0085 (5e-04)
	LDpred-funct-inf	-4e-04 (2e-04)	-1e-04 (2e-04)	-7e-04 (2e-04)
	LDpred-funct	0	0	0

(b)

# Causal variants	Model	Training sample size: 10,000 Diff. $R^2 (s.e.)$	20,000 Diff. $R^2 (s.e.)$	50,000 Diff. $R^2 (s.e.)$
1,000	P+T	-0.0165 (0.0035)	-0.0094 (0.0034)	-2e-04 (0.0033)
	LDpred	0	0	0
	P+T-funct-LASSO	0.0067 (0.0037)	0.0088 (0.0037)	0.0141 (0.0035)
	AnnoPred	0.0084 (0.0019)	0.0083 (0.0018)	0.0066 (0.0011)
	LDpred-funct-inf	-0.0321 (0.0017)	-0.0198 (0.0012)	0.0125 (6e-04)
	LDpred-funct	-0.0087 (0.0017)	0.0028 (0.0013)	0.0267 (8e-04)
2,000	P+T	-0.0352 (0.0036)	-0.0294 (0.0035)	-0.0254 (0.0036)
	LDpred	0	0	0
	P+T-funct-LASSO	-0.0146 (0.0039)	-0.0129 (0.0036)	-0.0121 (0.0037)
	AnnoPred	0.0074 (0.0025)	0.0091 (0.0019)	0.0045 (5e-04)
	LDpred-funct-inf	-0.0104 (0.0025)	-0.004 (0.0019)	0.0137 (5e-04)
	LDpred-funct	0.0019 (0.0026)	0.0078 (0.0019)	0.0213 (7e-04)
5,000	P+T	-0.048 (0.0024)	-0.0488 (0.0026)	-0.0466 (0.0031)
	LDpred	0	0	0
	P+T-funct-LASSO	-0.0283 (0.0026)	-0.03 (0.0028)	-0.0329 (0.0033)
	AnnoPred	0.0103 (0.0012)	0.0085 (8e-04)	0.0046 (5e-04)
	LDpred-funct-inf	0.0044 (0.0013)	0.0041 (7e-04)	0.0139 (4e-04)
	LDpred-funct	0.0067 (0.0013)	0.007 (7e-04)	0.0157 (5e-04)
10,000	P+T	-0.0493 (0.0022)	-0.0532 (0.0024)	-0.0551 (0.0031)
	LDpred	0	0	0
	P+T-funct-LASSO	-0.0348 (0.0024)	-0.0386 (0.0026)	-0.0454 (0.0033)
	AnnoPred	0.0118 (0.0013)	0.0094 (5e-04)	0.0051 (4e-04)
	LDpred-funct-inf	0.0111 (0.0012)	0.009 (4e-04)	0.0143 (5e-04)
	LDpred-funct	0.0107 (0.0013)	0.0089 (5e-04)	0.0136 (5e-04)

Supplementary Table 2: Differences between polygenic prediction methods in simulations using UK Biobank genotypes, for 4 values of the number of causal variants. We report results for P+T, LDpred, P+T-funct-LASSO, AnnoPred, LDpred-funct-inf and LDpred-funct in chromosome 1 simulations with 1,000 causal variants (very sparse architecture), 2,000 causal variants (sparse architecture), 5,000 causal variants (polygenic architecture) and 10,000 causal variants (very polygenic architecture). Results are averaged across 100 simulations. We report standard errors in parentheses. (a) Difference between R^2 for LDpred-funct vs. R^2 for each method. (b) Difference between R^2 for each method vs. R^2 for LDpred.

	Training sample size		
# Causal	10,000	20,000	50,000
1,000	0.03	0.1	1
2,000	0.03	0.1	1
5,000	0.03	0.1	1
10,000	0.1	0.3	1

Supplementary Table 3: Model parameter values for LDpred in simulations. We report the optimal value of p which is the fraction of non-zero effects in the prior.

# Causal		Training sample size 10,000	20,000	50,000
1,000	P+T	0.0001	0.0001	0.0001
	P+T-funct-LASSO HP SNP Set	0.1000	0.1000	0.3000
	P+T-funct-LASSO LP SNP Set	0.0100	0.0100	0.0100
2,000	P+T	0.0010	0.0010	0.0010
	P+T-funct-LASSO HP SNP Set	0.1000	0.1000	0.3000
	P+T-funct-LASSO LP SNP Set	0.0100	0.0100	0.0100
5,000	P+T	0.0100	0.0100	0.0100
	P+T-funct-LASSO HP SNP Set	0.3000	0.3000	0.3000
	P+T-funct-LASSO LP SNP Set	0.1000	0.1000	0.1000
10,000	P+T	0.1000	0.1000	0.0100
	P+T-funct-LASSO HP SNP Set	0.3000	0.3000	1.0000
	P+T-funct-LASSO LP SNP Set	0.1000	0.1000	0.1000

Supplementary Table 4: Model parameter values for P+T and P+T-funct-LASSO in simulations. We report the optimal p-value threshold for Pruning + Thresholding (P+T), optimal p-value threshold for P+T-funct-LASSO high prior SNP (HP) set and optimal p-value threshold for P+T-funct-LASSO low prior SNP (LP) set (p-values obtained from two-sided t-tests and the parameter is optimized over a range of values as described in the Methods section). Optimal R^2_{LD} values was 0.1.

	Training sample size		
# Causal	10,000	20,000	50,000
1,000	0.03	0.10	1.00
2,000	0.03	0.10	1.00
5,000	0.03	0.10	1.00
10,000	0.10	0.10	1.00

Supplementary Table 5: Model parameter values for AnnoPred in simulations. Model parameter values for AnnoPred in simulations. We report the optimal value of p, the proportion of causal variants in the AnnoPred model, for each number of causal variants and sample size simulated.

Method	Posterior mean effects	LD matrices	Total
P+T	NA	11	11
LDpred	4,268 (151)	3734	8,003
SBayesR	142 (9)	19200	19,342
P+T-func-LASSO	NA	11	11
AnnoPred	5,249 (87)	3734	8,983
LDpred-funct-inf	71 (10.47)	NA	71
LDpred-funct	71 (10.47)	NA	71

Supplementary Table 6: Average running time for all 7 methods. We report average running times in minutes for each method. We separately report the time to estimate posterior mean causal effect sizes, and the time to compute LD matrices (not applicable for LDpred-funct-inf and LD-pred-funct) (we do not include the time to compute polygenic risk scores, which is small in comparison and depends on the number of validation samples). LDpred-funct includes an additional step, the estimation of regularization weights, but this adds minimal additional computational cost (roughly 2 minutes). We estimated running times for chromosome 1 (roughly 10% of the genome) across 20 simulations for estimation of posterior mean effect sizes and one simulation for LD computation, and extrapolated the results to the whole genome. Thus, we report s.e. in parentheses for estimation of posterior mean effect sizes only. LDpred, AnnoPred, LDpred-funct-inf and LDpred-funct require a data coordination step (coordinating the summary statistics file, valditation genotypes and LD reference genotypes (in this case the same as the validation)), which required an additional 193 minutes for LDpred and AnnoPred and 141 minutes for LDpred-funct-inf and LDpred-funct. The running time for computation of the LD matrices for SBayesR is copied from ref. 9 (1.1M SNP set), as we did not repeat this computation.

# Causal variants	Model	Training sample size 10,000 Average $R^2 (s.e.)$	20,000 Average $R^2 (s.e.)$	50,000 Average $R^2 (s.e.)$
1,000	P+T	0.9371 (0.0282)	0.9806 (0.0294)	0.8189 (0.0486)
	LDpred	0.992 (0.0146)	0.947 (0.0083)	0.8521 (0.004)
	P+T-funct-LASSO	1.5051 (0.0589)	1.3703 (0.0386)	1.0151 (0.075)
	AnnoPred	0.8222 (0.0171)	0.7832 (0.0187)	0.761 (0.0154)
	LDpred-funct-inf	0.4708 (0.0025)	0.454 (0.002)	0.4345 (0.0024)
	LDpred-funct	0.9803 (6e-04)	0.9847 (4e-04)	0.9877 (4e-04)
2,000	P+T	0.7644 (0.0309)	0.791 (0.0257)	0.7976 (0.0209)
	LDpred	0.9688 (0.037)	0.9346 (0.0257)	0.8483 (0.0044)
	P+T-funct-LASSO	1.3572 (0.0382)	1.2138 (0.0544)	1.0448 (0.0284)
	AnnoPred	0.9258 (0.0084)	0.8849 (0.0089)	0.8136 (0.0025)
	LDpred-funct-inf	0.4656 (0.004)	0.457 (0.0028)	0.4396 (0.0021)
	LDpred-funct	0.9787 (0.001)	0.9837 (7e-04)	0.9882 (4e-04)
5,000	P+T	0.4546 (0.0207)	0.5954 (0.0172)	0.6728 (0.0158)
	LDpred	0.9984 (0.0067)	0.9671 (0.0071)	0.8538 (0.0044)
	P+T-funct-LASSO	0.8085 (0.0267)	0.8994 (0.012)	0.909 (0.0213)
	AnnoPred	0.949 (0.0034)	0.9092 (0.0031)	0.8128 (0.0024)
	LDpred-funct-inf	0.47 (0.0035)	0.4584 (0.0023)	0.4424 (0.0015)
	LDpred-funct	0.9776 (9e-04)	0.9839 (5e-04)	0.9881 (4e-04)
10,000	P+T	0.3196 (0.0136)	0.4655 (0.016)	0.586 (0.0116)
	LDpred	0.9903 (0.0156)	0.9449 (0.0059)	0.847 (0.0041)
	P+T-funct-LASSO	0.6824 (0.0182)	0.8142 (0.0182)	0.8178 (0.017)
	AnnoPred	0.9468 (0.0033)	0.9086 (0.0034)	0.811 (0.0021)
	LDpred-funct-inf	0.4654 (0.0028)	0.4528 (0.0025)	0.4365 (0.0024)
	LDpred-funct	0.9761 (7e-04)	0.9824 (6e-04)	0.9874 (4e-04)

Supplementary Table 7: Calibration of 6 polygenic prediction methods in simulations using UK Biobank genotypes, for 4 values of the number of causal variants. We report calibration slopes for P+T, LDpred, P+T-funct-LASSO, AnnoPred, LDpred-funct-inf and LDpred-funct in chromosome 1 simulations with 1,000 causal variants (very sparse architecture), 2,000 causal variants (sparse architecture), 5,000 causal variants (polygenic architecture) and 10,000 causal variants (very polygenic architecture). Results are averaged across 100 simulations.

# Causal variants	Model	Training sample size 10,000 Average $R^2 (s.e.)$	20,000 Average $R^2 (s.e.)$	50,000 Average $R^2 (s.e.)$
1,000	LDpred-funct-inf	0.1896 (0.0018)	0.2419 (0.0019)	0.3015 (0.0019)
	LDpred-funct-inf-5	0.208 (0.002)	0.2585 (0.002)	0.3104 (0.0019)
	LDpred-funct-inf-10	0.2101 (0.002)	0.261 (0.002)	0.3124 (0.002)
	LDpred-funct-inf-20	0.2116 (0.002)	0.263 (0.002)	0.314 (0.002)
	LDpred-funct-inf-30	0.2126 (0.002)	0.2638 (0.002)	0.315 (0.002)
	LDpred-funct-inf-40	0.2131 (0.002)	0.2644 (0.0021)	0.3157 (0.002)
	LDpred-funct-inf-50	0.2141 (0.002)	0.2652 (0.0021)	0.3161 (0.002)
	LDpred-funct-inf-60	0.2145 (0.0021)	0.2655 (0.0021)	0.3172 (0.002)
	LDpred-funct-inf-70	0.2157 (0.0021)	0.266 (0.0021)	0.317 (0.0021)
	LDpred-funct-inf-80	0.216 (0.002)	0.2665 (0.0021)	0.3173 (0.0021)
	LDpred-funct-inf-90	0.2164 (0.0021)	0.2667 (0.0021)	0.3176 (0.0021)
	LDpred-funct-inf-100	0.2165 (0.0021)	0.267 (0.0021)	0.3174 (0.0021)
2,000	LDpred-funct-inf	0.1900 (0.0015)	0.2458 (0.0015)	0.3057 (0.0016)
	LDpred-funct-inf-5	0.1994 (0.0016)	0.254 (0.0016)	0.3101 (0.0016)
	LDpred-funct-inf-10	0.2005 (0.0016)	0.2554 (0.0016)	0.3113 (0.0017)
	LDpred-funct-inf-20	0.2016 (0.0016)	0.2566 (0.0016)	0.3124 (0.0017)
	LDpred-funct-inf-30	0.2018 (0.0016)	0.2572 (0.0016)	0.3129 (0.0017)
	LDpred-funct-inf-40	0.2023 (0.0016)	0.2576 (0.0016)	0.3134 (0.0017)
	LDpred-funct-inf-50	0.2023 (0.0016)	0.2575 (0.0016)	0.3136 (0.0017)
	LDpred-funct-inf-60	0.2025 (0.0016)	0.258 (0.0017)	0.3137 (0.0017)
	LDpred-funct-inf-70	0.2027 (0.0016)	0.2579 (0.0017)	0.3135 (0.0017)
	LDpred-funct-inf-80	0.2031 (0.0016)	0.2583 (0.0017)	0.3133 (0.0017)
	LDpred-funct-inf-90	0.2028 (0.0016)	0.2579 (0.0017)	0.3134 (0.0018)
	LDpred-funct-inf-100	0.2031 (0.0016)	0.2582 (0.0017)	0.313 (0.0018)
5,000	LDpred-funct-inf	0.1872 (0.0012)	0.243 (0.0013)	0.3063 (0.0014)
	LDpred-funct-inf-5	0.1895 (0.0012)	0.2451 (0.0013)	0.3075 (0.0014)
	LDpred-funct-inf-10	0.1898 (0.0012)	0.2456 (0.0013)	0.3079 (0.0014)
	LDpred-funct-inf-20	0.1897 (0.0012)	0.2461 (0.0013)	0.3083 (0.0014)
	LDpred-funct-inf-30	0.1898 (0.0012)	0.2461 (0.0013)	0.3084 (0.0014)
	LDpred-funct-inf-40	0.1895 (0.0012)	0.2458 (0.0013)	0.3081 (0.0014)
	LDpred-funct-inf-50	0.1894 (0.0012)	0.2457 (0.0013)	0.3081 (0.0014)
	LDpred-funct-inf-60	0.1893 (0.0012)	0.2454 (0.0013)	0.3077 (0.0014)
	LDpred-funct-inf-70	0.1891 (0.0012)	0.245 (0.0013)	0.3073 (0.0014)
	LDpred-funct-inf-80	0.1888 (0.0012)	0.2447 (0.0013)	0.3071 (0.0014)
	LDpred-funct-inf-90	0.1885 (0.0012)	0.2444 (0.0013)	0.3066 (0.0014)
	LDpred-funct-inf-100	0.188 (0.0012)	0.244 (0.0013)	0.3062 (0.0014)
10,000	LDpred-funct-inf	0.1873 (0.0012)	0.2419 (0.0012)	0.3059 (0.0013)
	LDpred-funct-inf-5	0.1883 (0.0012)	0.2428 (0.0012)	0.3064 (0.0013)
	LDpred-funct-inf-10	0.1882 (0.0012)	0.2428 (0.0012)	0.3064 (0.0012)
	LDpred-funct-inf-20	0.1878 (0.0012)	0.2427 (0.0012)	0.3061 (0.0012)
	LDpred-funct-inf-30	0.1873 (0.0013)	0.2422 (0.0012)	0.3056 (0.0013)
	LDpred-funct-inf-40	0.187 (0.0013)	0.2418 (0.0012)	0.3053 (0.0012)
	LDpred-funct-inf-50	0.1865 (0.0012)	0.2414 (0.0012)	0.3049 (0.0013)
	LDpred-funct-inf-60	0.186 (0.0013)	0.2409 (0.0012)	0.3043 (0.0013)
	LDpred-funct-inf-70	0.1855 (0.0013)	0.2406 (0.0012)	0.3039 (0.0013)
	LDpred-funct-inf-80	0.1851 (0.0012)	0.2399 (0.0012)	0.3036 (0.0012)
	LDpred-funct-inf-90	0.1846 (0.0013)	0.2393 (0.0012)	0.3027 (0.0013)
	LDpred-funct-inf-100	0.1841 (0.0013)	0.2387 (0.0012)	0.3027 (0.0013)

Supplementary Table 8: Sensitivity of LDpred-funct results to number of bins used for regularization in simulations using UK Biobank genotypes. We report results with the number of posterior mean causal effect size bins used for regularization (K) set to 10, 20, 50 or 100. LDpred-funct-K denotes each respective value of K. We also report results for LDpred-funct-inf, which is identical to LDpred-funct with K set to 1. Results are averaged across 100 simulations. We report standard errors in parentheses.

# Causal variants	Model	Training sample size 10,000 Average $R^2 (s.e.)$	20,000 Average $R^2 (s.e.)$	50,000 Average $R^2 (s.e.)$
1,000	LDpred-funct-inf	0.1896 (0.0018)	0.2419 (0.0019)	0.3015 (0.0019)
	LDpred-funct	0.2131 (0.002)	0.2644 (0.0021)	0.3157 (0.002)
	LDpred-funct-inf-cheat	0.1926 (0.0018)	0.2456 (0.0019)	0.3074 (0.002)
	LDpred-funct-cheat	0.2221 (0.0021)	0.2714 (0.0022)	0.3228 (0.0021)
2,000	LDpred-funct-inf	0.1900 (0.0015)	0.2458 (0.0015)	0.3057 (0.0016)
	LDpred-funct	0.2023 (0.0016)	0.2576 (0.0016)	0.3134 (0.0017)
	LDpred-funct-inf-cheat	0.1943 (0.0015)	0.2498 (0.0016)	0.3108 (0.0016)
	LDpred-funct-cheat	0.2109 (0.0016)	0.2646 (0.0017)	0.3193 (0.0017)
5,000	LDpred-funct-inf	0.1872 (0.0012)	0.243 (0.0013)	0.3063 (0.0014)
	LDpred-funct	0.1895 (0.0012)	0.2458 (0.0013)	0.3081 (0.0014)
	LDpred-funct-inf-cheat	0.1928 (0.0013)	0.2479 (0.0013)	0.3102 (0.0014)
	LDpred-funct-cheat	0.1972 (0.0014)	0.252 (0.0013)	0.3121 (0.0014)
10,000	LDpred-funct-inf	0.1873 (0.0012)	0.2419 (0.0012)	0.3059 (0.0013)
	LDpred-funct	0.1870 (0.0013)	0.2418 (0.0012)	0.3053 (0.0012)
	LDpred-funct-inf-cheat	0.1937 (0.0012)	0.2474 (0.0012)	0.3097 (0.0012)
	LDpred-funct-cheat	0.194 (0.0013)	0.2482 (0.0013)	0.3096 (0.0013)

Supplementary Table 9: Accuracy of LDpred-funct method in simulations using UK Biobank genotypes under different BaselineLD estimates, for 4 values of the number of causal variants. LDpred-funct-cheat refers to a "cheating" version of LDpred-funct that utilized the true baseline-LD model parameters used to simulate the data. Results are averaged across 100 simulations.

# Causal variants	Model	Training sample size 10,000 Average $R^2 (s.e.)$	20,000 Average $R^2 (s.e.)$	50,000 Average $R^2 (s.e.)$
2,000	P+T	0.0634 (0.0015)	0.0912 (0.0019)	0.118 (0.0056)
	LDpred	0.0885 (0.0025)	0.1174 (0.0029)	0.1447 (0.0028)
	P+T-funct-LASSO	0.0695 (0.0022)	0.0988 (0.0021)	0.1213 (0.006)
	AnnoPred	0.0848 (0.0024)	0.1097 (0.0021)	0.134 (0.0023)
	LDpred-funct-inf	0.0759 (0.0019)	0.1033 (0.0021)	0.1384 (0.0023)
	LDpred-funct	0.0808 (0.0023)	0.1094 (0.0023)	0.1432 (0.0022)
5,000	P+T	0.0503 (0.0013)	0.0783 (0.0014)	0.1033 (0.0051)
	LDpred	0.0746 (0.0016)	0.1041 (0.0016)	0.1382 (0.0021)
	P+T-funct-LASSO	0.0578 (0.0015)	0.0869 (0.0015)	0.1087 (0.0056)
	AnnoPred	0.0772 (0.0016)	0.1031 (0.0025)	0.1334 (0.0019)
	LDpred-funct-inf	0.0744 (0.0014)	0.1032 (0.0016)	0.1375 (0.0018)
	LDpred-funct	0.0754 (0.0015)	0.105 (0.0017)	0.139 (0.0018)

Supplementary Table 10: Accuracy of 6 polygenic prediction methods in simulations with lower SNP-heritability ($h_g^2 = 0.25$) using UK Biobank genotypes, for 4 values of the number of causal variants. We report results for P+T, LDpred, P+T-funct-LASSO, AnnoPred, LDpred-funct-inf and LDpred-funct in chromosome 1 simulations with 1,000 causal variants (very sparse architecture), 2,000 causal variants (sparse architecture), 5,000 causal variants (polygenic architecture) and 10,000 causal variants (very polygenic architecture). Results are averaged across 20 simulations. We report standard errors in parentheses. For SBayesR simulations at N=10K, we obtained average prediction R^2 of 0.0625 (0.0086) and 0.0618 (0.0053) for 2,000, and 5,000 causal variants respectively. However, for all SBayesR simulations at N=20K and N=50K, we obtained average prediction R^2 of 0.0002 (0.0001) and $h_g^2 = 0.99$ in 20/20 simulations, perhaps because the algorithm failed to converge.

	Trait	h_g^2	Training N	Validation N (ancestry distribution)
1	Height	0.57	408092	24351 (40.3% Irish,59.7% Other)
2	Hair color	0.45	403024	24114 (40.3% Irish,59.7% Other)
3	Platelet count	0.40	395747	23616 (40.4% Irish,59.6% Other)
4	Bone mineral density	0.40	397274	23505 (40.4% Irish,59.6% Other)
5	Red blood cell count	0.32	396464	23644 (40.4% Irish,59.6% Other)
6	Age at menarche	0.31	214860	13737 (36.5% Irish,63.5% Other)
7	FEV1 FVC ratio	0.31	331786	19457 (39.5% Irish,60.5% Other)
8	Body mass index	0.31	407667	24322 (40.3% Irish,59.7% Other)
9	RBC distribution width	0.29	394258	23518 (40.3% Irish,59.7% Other)
10	Forced vital capacity	0.27	331786	19457 (39.5% Irish,60.5% Other)
11	Eosinophil count	0.27	391787	23377 (40.3% Irish,59.7% Other)
12	White blood cell count	0.27	395835	23634 (40.4% Irish,59.6% Other)
13	Systolic Blood pressure	0.27	376437	22531 (40.1% Irish,59.9% Other)
14	Waist hip ratio	0.21	408196	24354 (40.3% Irish,59.7% Other)

Supplementary Table 11: List of 14 UK Biobank quantitative traits. We list the training sample size and validation sample size for each trait. h_g^2 estimates are obtained using BOLT-LMM v2.3 using the training data set.

	Trait	h_g^2	Training		Validation	
			N	Prevalence	N (ancestry distribution)	Prevalence
1	Balding Type I	0.32	186,506	0.32	10,171 (45.9% Irish,54.1% Other)	0.35
2	Tanning	0.23	400,721	0.39	23,947 (40.4% Irish,59.6% Other)	0.61
3	College Education	0.20	405,140	0.31	24,085 (40.4% Irish,59.6% Other)	0.50
4	Hyperthension	0.18	408,323	0.27	24,368 (40.3% Irish,59.7% Other)	0.24
5	Cardiovascular Diseases	0.16	408,963	0.32	24,435 (40.3% Irish,59.7% Other)	0.29
6	Morning Person	0.14	365,245	0.37	22,188 (40.2% Irish,59.8% Other)	0.58
7	Eczema	0.12	408,454	0.23	24,374 (40.4% Irish,59.6% Other)	0.23

Supplementary Table 12: List of 7 UK Biobank binary traits. We list the training sample size, validation sample size and prevalence for each trait. h_g^2 estimates are obtained using BOLT-LMM v2.3 using the training data set.

	Trait	Training N	h_g^2	c	bins
1	Height	408092	0.57	0.45	100
2	Hair color	403024	0.45	0.22	100
3	Platelet count	395747	0.40	0.29	88
4	Bone mineral density	397274	0.40	0.26	87
5	Red blood cell count	396464	0.32	0.21	70
6	Age at menarche	214860	0.31	0.20	40
7	FEV1 FVC ratio	331786	0.31	0.24	56
8	Body mass index	407667	0.31	0.27	70
9	RBC distribution width	394258	0.29	0.20	63
10	Eosinophil count	391787	0.27	0.18	60
11	Forced vital capacity	331786	0.27	0.22	50
12	White blood cell count	395835	0.27	0.21	60
13	Systolic Blood pressure	376437	0.27	0.21	56
14	Waist hip ratio	408196	0.21	0.15	48
1	Balding type I	186506	0.32	0.11	31
2	Tanning ability	400721	0.23	0.09	53
3	College Education	405140	0.20	0.15	45
4	Hyperthension	408323	0.18	0.14	41
5	Cardiovascular Diseases	408963	0.16	0.12	37
6	Morning Person	365245	0.14	0.11	29
7	Eczema	408454	0.12	0.09	27

Supplementary Table 13: Parameter values for 21 UK Biobank traits. The 14 quantitative traits are listed first, followed by the 7 binary traits. For each trait, we list the training sample size, h_g^2 estimate (from BOLT-LMM v2.3; used by LDpred, LDpred-funct-inf and LDpred-funct), the c parameter (used by LDpred-funct-inf and LDpred-funct) and number of bins for LDpred-funct.

	Trait	h_g^2	P+T	LDpred	SBayesR	P+T-funct-LASSO	AnnoPred	LDpred-funct -inf	LDpred-funct
1	Height	0.575	0.3481 (0.0145)	0.3820 (0.0204)	0.3820 (0.0197)	0.3684 (0.0172)	0.4063 (0.0276)	0.4003 (0.0196)	0.4131 (0.0261)
2	Hair color	0.446	0.2418 (0.0794)	0.2507 (0.1024)	0.3241 (0.1236)	0.2413 (0.0874)	0.2616 (0.1095)	0.2638 (0.1059)	0.3258 (0.1305)
3	Platelet count	0.401	0.2044 (0.0228)	0.2349 (0.0227)	0.2338 (0.0264)	0.2187 (0.0245)	0.2333 (0.0221)	0.2290 (0.0202)	0.2428 (0.0275)
4	Bone mineral density	0.398	0.1906 (0.0190)	0.2096 (0.0216)	0.2176 (0.0193)	0.2024 (0.0206)	0.2281 (0.0210)	0.2107 (0.0186)	0.2227 (0.0248)
5	Red blood cell count	0.319	0.1244 (0.0143)	0.1494 (0.0158)	0.1523 (0.0167)	0.1309 (0.0149)	0.1676 (0.0158)	0.1568 (0.0141)	0.1652 (0.0207)
6	Age at menarche	0.313	0.0716 (0.0054)	0.1078 (0.0100)	0.1057 (0.0098)	0.0873 (0.0066)	0.1113 (0.0103)	0.1054 (0.0090)	0.1078 (0.0185)
7	FEV1 FVC ratio	0.309	0.1012 (0.0077)	0.1238 (0.0095)	0.1348 (0.0098)	0.1124 (0.0076)	0.1417 (0.0099)	0.1289 (0.0090)	0.1317 (0.0172)
8	Body mass index	0.307	0.1071 (0.0044)	0.1414 (0.0076)	0.1402 (0.0072)	0.1186 (0.0059)	0.1519 (0.0079)	0.1491 (0.0073)	0.1481 (0.0152)
9	RBC distribution width	0.286	0.1190 (0.0141)	0.1302 (0.0147)	0.1399 (0.0157)	0.1299 (0.0177)	0.1458 (0.0147)	0.1394 (0.0145)	0.1496 (0.0203)
10	Eosinophil count	0.274	0.1109 (0.0102)	0.1259 (0.0133)	0.1288 (0.0131)	0.1169 (0.0102)	0.1391 (0.0144)	0.1342 (0.0130)	0.1410 (0.0194)
11	Forced vital capacity	0.274	0.0763 (0.0054)	0.1083 (0.0072)	0.1070 (0.0069)	0.0893 (0.0053)	0.1196 (0.0074)	0.1153 (0.0068)	0.1147 (0.0152)
12	White blood cell count	0.273	0.0964 (0.0069)	0.1145 (0.0095)	0.1256 (0.0095)	0.1085 (0.0093)	0.1325 (0.0094)	0.1244 (0.0090)	0.1274 (0.0160)
13	Systolic Blood pressure	0.267	0.0827 (0.0055)	0.1066 (0.0071)	0.1084 (0.0069)	0.0967 (0.0053)	0.1195 (0.0071)	0.1135 (0.0066)	0.1136 (0.0138)
14	Waist hip ratio	0.210	0.0567 (0.0039)	0.0782 (0.0062)	0.0782 (0.0064)	0.0646 (0.0046)	0.0855 (0.0069)	0.0786 (0.0051)	0.0806 (0.0117)
15	Average	0.330	0.1379 (0.002)	0.1617 (0.0098)	0.1699 (0.0108)	0.1490 (0.008)	0.1745 (0.0103)	0.1678 (0.0098)	0.1774 (0.0113)

Supplementary Table 14: Accuracy of 7 polygenic prediction methods across 14 UK Biobank quantitative traits. We report results for P+T, LDpred, SBayesR, P+T-funct-LASSO, AnnoPred, LDpred-funct-inf and LDpred-funct. Optimal parameters for each method are reported in Supplementary Table 19, Supplementary Table 18, Supplementary Table 21 and Supplementary Table 13. We report block jackknife standard error over 200 equally sized blocks of adjacent SNPs.

	Trait	h_g^2	P+T	LDpred	SBayesR	P+T-funct-LASSO	AnnoPred	LDpred-funct-inf	LDpred-funct
1	Balding type I	0.323	0.1164 (0.0129)	0.1326 (0.0171)	0.1272 (0.0197)	0.1272 (0.0162)	0.1461 (0.0211)	0.1078 (0.0135)	0.1237 (0.0245)
2	Tanning ability	0.235	0.1293 (0.0445)	0.1278 (0.0670)	0.1688 (0.0678)	0.1298 (0.0467)	0.1199 (0.0633)	0.1219 (0.0626)	0.1788 (0.0771)
3	College Education	0.198	0.0611 (0.0028)	0.0687 (0.0063)	0.0711 (0.0061)	0.0636 (0.0029)	0.0694 (0.0064)	0.0770 (0.0061)	0.0792 (0.0113)
4	Hyperthension	0.179	0.0405 (0.0028)	0.0537 (0.0046)	0.0575 (0.0044)	0.0467 (0.0032)	0.0553 (0.0047)	0.0527 (0.0044)	0.0523 (0.0093)
5	Cardiovascular Diseases	0.160	0.0275 (0.0020)	0.0424 (0.0038)	0.0464 (0.0039)	0.0335 (0.0025)	0.0451 (0.0039)	0.0432 (0.0037)	0.0429 (0.0085)
6	Morning Person	0.137	0.0265 (0.0021)	0.0382 (0.0033)	0.0382 (0.0031)	0.0308 (0.0025)	0.0395 (0.0032)	0.0413 (0.0032)	0.0409 (0.0087)
7	Eczema	0.118	0.0149 (0.0016)	0.0273 (0.0032)	0.0254 (0.0029)	0.0210 (0.0025)	0.0307 (0.0034)	0.0279 (0.0031)	0.0284 (0.0070)
8	Average	0.190	0.0594 (0.0015)	0.0701 (0.0103)	0.0764 (0.0103)	0.0647 (0.0073)	0.0723 (0.0100)	0.0674 (0.0095)	0.0780 (0.0116)

Supplementary Table 15: Accuracy of 7 polygenic prediction methods across 7 UK Biobank binary traits. We report results for P+T, LDpred, SBayesR, P+T-funct-LASSO, AnnoPred, LDpred-funct-inf and LDpred-funct. Optimal parameters for each method are reported in Supplementary Table 19, Supplementary Table 18, Supplementary Table 21 and Supplementary Table 13. We report block jackknife standard error over 200 equally sized blocks of adjacent SNPs.

Phenotype	Average difference vs LDpred	Average difference vs LDpred-funct
P+T	-0.0194 (0.0081)	-0.0325 (0.0094)
LDPRED	0.000 (0.0000)	-0.0131 (0.0034)
SBayesR	0.0067 (0.0031)	-0.0064 (0.0031)
P+T-LASSO	-0.0108 (0.003)	-0.0239 (0.004)
Annopred	0.0093 (0.002)	-0.0038 (0.0034)
LDPRED-funct-inf	0.0032 (0.0019)	-0.0099 (0.0026)
LDPRED-funct	0.0131 (0.0034)	0.000 (0.0000)

Supplementary Table 16: Average absolute differences between polygenic prediction methods across 21 UK Biobank traits. We report results for P+T, LDpred, SBayesR, P+T-funct-LASSO, AnnoPred, LDpred-funct-inf and LDpred-funct. We report the difference between prediction R^2 for each method vs. prediction R^2 for LDpred, and difference between prediction R^2 for each method vs. prediction R^2 for LDpred-funct. Block-jackknife standard errors on the differences are reported in parentheses.

	Method	Average R^2
1	P+T	0.1112
2	LDpred	0.1311
3	SBayesR	0.1379
4	P+T-funct-LASSO	0.1203
5	AnnoPred	0.1405
6	LDpred-funct-inf	0.1343
7	LDpred-funct	0.1443
8	LDpred-inf	0.1126
9	LDpred (without excluding long-range LD regions)	0.0839
10	LDpred (typed SNPs only)	0.1299
11	LDpred-funct-inf (typed SNPs only)	0.1124
12	LDpred-funct (typed SNPs only)	0.1198
13	SBayesR (2.9M)	0.1227
14	P+T-funct-LASSO-weighted	0.1231
15	P+T-funct-LASSO (5%)	0.1219
16	LDpred-funct-inf (meta31)	0.1307
17	P+T-LASSO (random)	0.1178
18	AnnoPred (random)	0.1288
19	LDpred-funct-inf (random)	0.1153
20	LDpred-funct (random)	0.1271
21	LDpred-inf + sparsity	0.1282
22	LDpred-funct-inf (baseline)	0.1312
23	LDpred-funct (baseline)	0.1415
24	LDpred-funct-inf (constant prior)	0.1241
25	LDpred-funct (constant prior)	0.1383
26	LDpred-funct-inf(QCfilters)	0.1339
27	LDpred-funct-inf(UK10K)	0.1354
28	LDpred-funct-inf(UK10K, baseline-LD+LDAK)	0.1350
29	LDpred-funct-inf (Baseline-LD v2.1)	0.1360
30	LDpred-funct (Baseline-LD v2.1)	0.1469

Supplementary Table 17: Accuracy of secondary polygenic prediction methods across 21 UK Biobank traits. Rows 1-7 correspond to average prediction R^2 across 21 UK Biobank traits for each method. Row 8 correspond to the average prediction R^2 from LDpred-inf. Row 9 correspond to the average prediction R^2 from LDpred that includes SNPs from long-range LD regions. Rows 10-12 are methods that analyze only genotyped SNPs (601,728 genotyped SNPs after QC). Row 13 correspond to the average prediction R^2 from SBayesR using 2.9M SNPs (SNP set described in Methods). Rows 14-15 are slightly modified versions of P+T-funct-LASSO. Row 16 uses baseline-LD model functional enrichments that were meta-analyzed across 31 traits. Rows 17-20 report. avg. prediction R^2 functionally informed methods, P+T-funct-LASSO, AnnoPred, LDpred-funct-inf and LDpred-funct using a set of 75 random annotations. Row 21 corresponds to avg. prediction R^2 of LDpred-inf with sparsity (i.e. LDpred-funct with no functional annotations except the annotation containing all SNPs). Row 22-23 uses the baseline model, instead of the baseline-LD model. Rows 24-25 report avg. prediction R^2 for LDpred-funct-inf and LDpred-funct with priors replaced by indicator function that gives a constant prior to SNPs with predicted $h^2 > 0$ and sets effect sizes to zero for SNPs with $h^2 < 0$. Row 26 restricts the baseline-LD model to the 6,334,603 SNPs that passed QC filters and were used for prediction. Row 27 infers baseline-LD model parameters using UK10K SNPs, instead of 1000 Genomes SNPs. Row 28 uses UK10K SNPs and uses the baseline-LD+LDAK model, instead of the baseline-LD model. Row 29-30 corresponds to the average prediction R^2 for LDpred-funct-inf and LDpred-funct using baseline-LD model v2.1 (instead of baseline-LD model v1.1, which is used in our main analyses).

	Trait	h_g^2	p
1	Height	0.57	0.3000
2	Hair color	0.45	0.3000
3	Platelet count	0.40	0.1000
4	Bone mineral density	0.40	0.1000
5	Balding type I	0.32	0.0300
6	Red blood cell count	0.32	0.1000
7	Age at menarche	0.31	0.0300
8	FEV1 FVC ratio	0.31	0.1000
9	Body mass index	0.31	0.1000
10	RBC distribution width	0.29	0.1000
11	Forced vital capacity	0.27	0.0300
12	Eosinophil count	0.27	0.0300
13	White blood cell count	0.27	0.1000
14	Systolic Blood pressure	0.27	0.1000
15	Tanning ability	0.23	0.1000
16	Waist hip ratio	0.21	0.0300
17	College Education	0.20	0.1000
18	Hyperthension	0.18	0.0300
19	Cardiovascular Diseases	0.16	0.0300
20	Morning Person	0.14	0.0100
21	Eczema	0.12	0.0100

Supplementary Table 18: Model parameter values for LDpred applied to 21 UK Biobank traits. h_g^2 estimate (from BOLT-LMM v2.3), p is the fraction of non-zero effects in the prior.

	Phenotype	h_g^2	P+T	P-values threshold for P+T-funct-LASSO HP SNP set	P+T-funct-LASSO LP SNP set
1	Height	0.57	0.0100	0.30	0.10
2	Hair color	0.45	0.0010	0.30	0.01
3	Platelet count	0.40	0.0010	0.10	0.10
4	Bone mineral density	0.40	0.0010	0.10	0.10
5	Balding type I	0.32	0.0010	0.10	0.01
6	Red blood cell count	0.32	0.0010	0.10	0.10
7	Age at menarche	0.31	0.0100	0.10	0.10
8	FEV1 FVC ratio	0.31	0.0010	0.10	0.10
9	Body mass index	0.31	0.1000	0.30	0.10
10	RBC distribution width	0.29	0.0010	0.10	0.01
11	Forced vital capacity	0.27	0.0010	0.10	0.10
12	Eosinophil count	0.27	0.0010	0.10	0.10
13	White blood cell count	0.27	0.0100	0.10	0.10
14	Systolic Blood pressure	0.27	0.0010	0.10	0.10
15	Tanning ability	0.23	0.0001	0.10	0.01
16	Waist hip ratio	0.21	0.0100	0.10	0.10
17	College Education	0.20	1.0000	0.30	0.30
18	Hyperthension	0.18	0.0100	0.10	0.01
19	Cardiovascular Diseases	0.16	0.0010	0.10	0.01
20	Morning Person	0.14	0.0100	0.10	0.10
21	Eczema	0.12	0.0001	0.10	0.01

Supplementary Table 19: Model parameter values for P+T and P+T-funct-LASSO in 21 UK Biobank traits. We report the optimal p-value threshold for Pruning + Thresholding (P+T), optimal p-value threshold for P+T-funct-LASSO high prior SNP (HP) set and optimal p-value threshold for P+T-funct-LASSO low prior SNP (LP) set (p-values obtained from two-sided t-tests and the parameter is optimized over a range of values as described in the Methods section). Optimal R^2_{LD} values was 0.1.

	Phenotype	h_g^2	Absolute difference
1	Height	0.575	0.0343 (0.0048)
2	Hair color	0.446	0.0238 (0.0136)
3	Platelet count	0.401	0.0352 (0.0045)
4	Bone mineral density	0.398	0.0268 (0.0031)
5	Balding type I	0.323	0.0230 (0.0031)
6	Red blood cell count	0.319	0.0325 (0.0042)
7	Age at menarche	0.313	0.0151 (0.0027)
8	FEV1 FVC ratio	0.309	0.0202 (0.0030)
9	Body mass index	0.307	0.0126 (0.0019)
10	RBC distribution width	0.286	0.0388 (0.0074)
11	Forced vital capacity	0.274	0.0198 (0.0021)
12	Eosinophil count	0.274	0.0388 (0.0062)
13	White blood cell count	0.273	0.0239 (0.0037)
14	Systolic Blood pressure	0.267	0.0155 (0.0021)
15	Tanning ability	0.235	0.0394 (0.0183)
16	Waist hip ratio	0.210	0.0142 (0.0018)
17	College Education	0.198	0.0052 (0.0028)
18	Hyperthension	0.179	0.0074 (0.0013)
19	Cardiovascular Diseases	0.160	0.0060 (0.0012)
20	Morning Person	0.137	0.0042 (0.0015)
21	Eczema	0.118	0.0073 (0.0010)
22	Average across traits	0.286	0.0211 (0.0020)

Supplementary Table 20: Difference between LDpred-funct-inf and LDpred-inf. LDpred-funct-inf significantly outperforms LDpred-inf ($P < 10^{-20}$ for difference using one-sided z-test based on block-jackknife standard error)

	Trait	h_g^2	p
1	Height	0.57	0.3000
2	Hair color	0.45	1.0000
3	Platelet count	0.40	0.3000
4	Bone mineral density	0.40	0.3000
5	Balding type I	0.32	0.0300
6	Red blood cell count	0.32	0.3000
7	Age at menarche	0.31	0.1000
8	FEV1 FVC ratio	0.31	0.1000
9	Body mass index	0.31	0.1000
10	RBC distribution width	0.29	0.3000
11	Forced vital capacity	0.27	0.1000
12	Eosinophil count	0.27	0.1000
13	White blood cell count	0.27	0.1000
14	Systolic Blood pressure	0.27	0.3000
15	Tanning ability	0.23	0.3000
16	Waist hip ratio	0.21	0.1000
17	College Education	0.20	0.3000
18	Hyperthension	0.18	0.1000
19	Cardiovascular Diseases	0.16	0.1000
20	Morning Person	0.14	0.1000
21	Eczema	0.12	0.1000

Supplementary Table 21: Model parameter values for AnnoPred applied to 21 UK Biobank traits. We report the h_g^2 estimate (from BOLT-LMM v2.3) and the optimal value of p, the proportion of causal variants in the AnnoPred model.

	Trait	h_g^2	LDpred-funct-inf	Validation sample size 1000	2000	5000	10000	ALL
1	Height	0.57	0.4003	0.3970	0.4048	0.4108	0.4122	0.4131
2	Hair color	0.45	0.2638	0.2957	0.3010	0.3052	0.3081	0.3258
3	Platelet count	0.40	0.2290	0.2469	0.2425	0.2451	0.2432	0.2428
4	Bone mineral density	0.40	0.2107	0.2350	0.2300	0.2274	0.2242	0.2227
5	Balding type I	0.32	0.1078	0.1280	0.1227	0.1233	0.1233	0.1237
6	Red blood cell count	0.32	0.1568	0.1692	0.1643	0.1678	0.1642	0.1652
7	Age at menarche	0.31	0.1054	0.1143	0.1128	0.1121	0.1114	0.1078
8	FEV1 FVC ratio	0.31	0.1289	0.1365	0.1357	0.1327	0.1348	0.1317
9	Body mass index	0.31	0.1491	0.1530	0.1477	0.1522	0.1520	0.1481
10	RBC distribution width	0.29	0.1394	0.1513	0.1458	0.1528	0.1546	0.1496
11	Forced vital capacity	0.27	0.1153	0.1265	0.1165	0.1118	0.1123	0.1147
12	Eosinophil count	0.27	0.1342	0.1464	0.1415	0.1416	0.1432	0.1410
13	White blood cell count	0.27	0.1244	0.1325	0.1280	0.1284	0.1288	0.1274
14	Systolic Blood pressure	0.27	0.1135	0.1200	0.1109	0.1141	0.1109	0.1136
15	Tanning ability	0.23	0.1219	0.1521	0.1582	0.1786	0.1796	0.1788
16	Waist hip ratio	0.21	0.0786	0.0919	0.0842	0.0822	0.0801	0.0806
17	College Education	0.20	0.0770	0.0775	0.0771	0.0739	0.0717	0.0792
18	Hyperthension	0.18	0.0527	0.0554	0.0572	0.0542	0.0528	0.0523
19	Cardiovascular Diseases	0.16	0.0432	0.0494	0.0486	0.0432	0.0428	0.0429
20	Morning Person	0.14	0.0413	0.0417	0.0427	0.0383	0.0366	0.0409
21	Eczema	0.12	0.0279	0.0335	0.0313	0.0290	0.0276	0.0284
22	Average across traits	0.29	0.1343	0.1454	0.1430	0.1440	0.1435	0.1443

Supplementary Table 22: Sensitivity of LDpred-funct results to number of validation samples across 21 UK Biobank traits. We report results with the number of validation samples set to 1,000, 2,000, 5,000, 10,000 (the number of regularization bins is proportional to the number of validation samples; see Equation 6. Results are averaged across 100 random subsets of each size. ALL denotes results of LDpred-funct using the total number of validation samples (reported in Table Supplementary Table 11). We also report results for LDpred-funct-inf, which is equivalent to LDpred-funct in the limit of a very small number of validation samples.

	Phenotype	h_g^2	LDpred-funct-inf	LDpred-funct	LDpred-funct (1K regularization weights)
1	Height	0.57	0.4003	0.4131	0.4130
2	Hair color	0.45	0.2638	0.3258	0.3258
3	Platelet count	0.40	0.2290	0.2428	0.2427
4	Bone mineral density	0.40	0.2107	0.2227	0.2226
5	Balding type I	0.32	0.1078	0.1237	0.1238
6	Red blood cell count	0.32	0.1568	0.1652	0.1653
7	Age at menarche	0.31	0.1054	0.1078	0.1080
8	FEV1 FVC ratio	0.31	0.1289	0.1317	0.1317
9	Body mass index	0.31	0.1491	0.1481	0.1480
10	RBC distribution width	0.29	0.1394	0.1496	0.1497
11	Forced vital capacity	0.27	0.1153	0.1147	0.1147
12	Eosinophil count	0.27	0.1342	0.1410	0.1410
13	White blood cell count	0.27	0.1244	0.1274	0.1274
14	Systolic Blood pressure	0.27	0.1135	0.1136	0.1136
15	Tanning ability	0.23	0.1219	0.1788	0.1787
16	Waist hip ratio	0.21	0.0786	0.0806	0.0806
17	College Education	0.20	0.0770	0.0792	0.0793
18	Hyperthension	0.18	0.0527	0.0523	0.0524
19	Cardiovascular Diseases	0.16	0.0432	0.0429	0.0429
20	Morning Person	0.14	0.0413	0.0409	0.0410
21	Eczema	0.12	0.0279	0.0284	0.0284
22	Average across traits	0.29	0.1343	0.1443	0.1443

Supplementary Table 23: Sensitivity of LDpred-funct results to using only 1,000 samples to estimate regularization weights across 21 UK Biobank traits. We report results using only 1K of the 22K validation samples to estimate regularization weights and the remaining validation samples to compute predictions.

	Phenotype	409K training samples	404K training samples	difference	S.E.	P-value
1	Height	0.4131	0.4132	-0.0001	0.0216	0.9963
2	Hair color	0.3258	0.3196	0.0062	0.1830	0.9734
3	Platelet count	0.2428	0.2451	-0.0023	0.0248	0.9261
4	Bone mineral density	0.2227	0.2231	-0.0004	0.0212	0.9812
5	Balding type I	0.1237	0.1222	0.0015	0.0146	0.9127
6	Red blood cell count	0.1652	0.1676	-0.0024	0.0196	0.9066
7	Age at menarche	0.1078	0.1071	0.0007	0.0120	0.9535
8	FEV1 FVC ratio	0.1317	0.1310	0.0007	0.0122	0.9542
9	Body mass index	0.1481	0.1477	0.0004	0.0085	0.9625
10	RBC distribution width	0.1496	0.1488	0.0008	0.0171	0.9627
11	Forced vital capacity	0.1147	0.1144	0.0003	0.0081	0.9705
12	Eosinophil count	0.1410	0.1389	0.0021	0.0154	0.8915
13	White blood cell count	0.1274	0.1278	-0.0004	0.0097	0.9671
14	Systolic Blood pressure	0.1136	0.1134	0.0002	0.0076	0.9790
15	Tanning ability	0.1788	0.1822	-0.0034	0.1079	0.9749
16	Waist hip ratio	0.0806	0.0799	0.0007	0.0066	0.9276
17	College Education	0.0792	0.0784	0.0008	0.0066	0.9035
18	Hyperthension	0.0523	0.0527	-0.0004	0.0046	0.9307
19	Cardiovascular Diseases	0.0429	0.0426	0.0003	0.0038	0.9580
20	Morning Person	0.0409	0.0404	0.0005	0.0037	0.8925
21	Eczema	0.0284	0.0288	-0.0004	0.0036	0.9336
22	Average across traits	0.1443	0.1440	0.0003	0.0142	0.9831

Supplementary Table 24: Sensitivity of LDpred-funct results to using 5,000 samples removed from the set of training samples to estimate regularization weights across 21 UK Biobank traits. We report results using 5K samples omitted from the set of training samples (now reduced to 404K) to estimate regularization weights and the full set of 22K validation samples to compute predictions. Across 21 UK Biobank traits, we observed a 0.2% decrease in average prediction R^2 (0.1440 vs. 0.1443), and the decrease was not statistically significant (P=0.98 for difference using one-sided z-test based on block-jackknife standard error).

	Trait	h_g^2	P+T	LDpred	SBayesR	P+T-funct-LASSO	AnnoPred	LDpred -funct-inf	LDpred -funct
1	Height	0.575	0.2228	0.7595	1.0335	0.3034	0.7374	0.7367	0.9938
2	Hair color	0.446	0.2505	0.7254	0.9909	0.3058	0.7118	0.7182	0.9920
3	Platelet count	0.401	0.2429	0.8451	0.9645	0.3423	0.7678	0.8115	0.9895
4	Bone mineral density	0.398	0.2871	0.8192	0.9604	0.3477	0.8142	0.8246	0.9865
5	Balding type I	0.323	0.3693	0.8994	1.9855	0.5050	0.8034	0.8781	0.9776
6	Red blood cell count	0.319	0.2898	0.8583	0.9721	0.3458	0.8481	0.8202	0.9822
7	Age at menarche	0.313	0.1990	1.0227	0.6183	0.3430	0.8880	0.8706	0.9782
8	FEV1 FVC ratio	0.309	0.3021	0.8843	1.0014	0.3593	0.8591	0.8527	0.9740
9	Body mass index	0.307	0.1687	0.9138	1.0338	0.3541	0.8839	0.8599	0.9813
10	RBC distribution width	0.286	0.2839	0.8399	0.9454	0.4189	0.8287	0.8123	0.9833
11	Forced vital capacity	0.274	0.2237	0.9085	1.0890	0.3783	0.9106	0.8665	0.9770
12	Eosinophil count	0.274	0.2781	0.9082	0.9874	0.3298	0.8167	0.8518	0.9830
13	White blood cell count	0.273	0.2352	0.9033	1.0254	0.3707	0.8347	0.8538	0.9793
14	Systolic Blood pressure	0.267	0.2200	0.9050	1.0063	0.3637	0.9064	0.8453	0.9808
15	Tanning ability	0.235	0.2437	0.8312	1.8516	0.2873	0.7384	0.8292	0.9905
16	Waist hip ratio	0.210	0.2057	0.8453	0.9891	0.3344	0.8404	0.8500	0.9758
17	College Education	0.198	0.1345	1.0159	2.2891	0.2610	0.8747	0.8520	0.9728
18	Hyperthension	0.179	0.2140	0.9817	2.1853	0.3557	0.8328	0.8077	0.9710
19	Cardiovascular Diseases	0.160	0.1213	0.9376	2.1111	0.3296	0.8329	0.7953	0.9643
20	Morning Person	0.137	0.2158	1.0803	2.1999	0.3720	0.9299	0.8751	0.9651
21	Eczema	0.118	0.1752	0.7496	2.1296	0.4971	0.8271	0.7611	0.9634
22	Average across traits	0.286	0.2325	0.8873	1.3509	0.3574	0.8327	0.8273	0.9791

Supplementary Table 25: Calibration comparison for the 7 methods applied to 21 UK Biobank traits. We report calibration slopes for each method, where a value close to 1 respresents a well calibrated prediction.

	Trait	LDpred-funct-inf	LDpred-funct-10	LDpred-funct-20	LDpred-funct-50	LDpred-funct-75	LDpred-funct-100
1	Height	0.4003	0.4113	0.4116	0.4126	0.4127	0.4128
2	Hair color	0.2624	0.2998	0.3059	0.3174	0.3199	0.3290
3	Platelet count	0.2315	0.2445	0.2453	0.2445	0.2446	0.2448
4	Bone mineral density	0.2137	0.2266	0.2266	0.2271	0.2265	0.2256
5	Balding type I	0.1075	0.1217	0.1235	0.1220	0.1198	0.1185
6	Red blood cell count	0.1571	0.1651	0.1655	0.1660	0.1660	0.1649
7	Age at menarche	0.1082	0.1118	0.1116	0.1122	0.1112	0.1070
8	FEV1 FVC ratio	0.1311	0.1353	0.1348	0.1343	0.1336	0.1315
9	Body mass index	0.1508	0.1501	0.1504	0.1494	0.1481	0.1473
10	RBC distribution width	0.1421	0.1527	0.1535	0.1535	0.1530	0.1517
11	Forced vital capacity	0.1145	0.1160	0.1155	0.1145	0.1128	0.1118
12	Eosinophil count	0.1335	0.1425	0.1422	0.1415	0.1406	0.1395
13	White blood cell count	0.1239	0.1278	0.1284	0.1276	0.1266	0.1261
14	Systolic Blood pressure	0.1114	0.1129	0.1119	0.1118	0.1108	0.1105
15	Tanning ability	0.1229	0.1716	0.1794	0.1818	0.1873	0.1892
16	Waist hip ratio	0.0793	0.0818	0.0810	0.0804	0.0798	0.0782
17	College Education	0.0716	0.0720	0.0731	0.0731	0.0748	0.0739
18	Hyperthension	0.0523	0.0542	0.0541	0.0528	0.0521	0.0519
19	Cardiovascular Diseases	0.0423	0.0437	0.0433	0.0421	0.0410	0.0410
20	Morning Person	0.0372	0.0372	0.0366	0.0359	0.0349	0.0340
21	Eczema	0.0274	0.0278	0.0275	0.0271	0.0274	0.0258
22	Average across traits	0.1343	0.1432	0.1439	0.1442	0.1440	0.1436

Supplementary Table 26: Sensitivity of LDpred-funct results to number of bins used for regularization across 21 UK Biobank traits. We report results with the number of posterior mean causal effect size bins used for regularization (K) set to 10, 20, 50, 75 or 100. LDpred-funct-K denotes each respective value of K. We also report results for LDpred-funct-inf, which is identical to LDpred-funct with K set to 1.

Data Set	Training N	P+T	LDpred	P+T-funct -LASSO	AnnoPred	LDpred-funct-inf	LDpred-funct
UK Biobank interim release	113660	0.2289	0.2265	0.2609	0.2530	0.2465	0.2506
UK Biobank	408092	0.3448	0.3823	0.3644	0.4063	0.4010	0.4152
23andMe	698430	0.2997	0.2953	0.3141	0.3166	0.3203	0.3442
Meta-analysis of UK Biobank and 23andMe	1107430	0.3710	0.3990	0.3778	0.4189	0.4193	0.4310
Fixed-effect meta-analysis	1107430	0.3687	0.3688	0.3663	0.3982	0.3991	0.4142

Supplementary Table 27: Accuracy of 6 prediction methods in height meta-analysis of UK Biobank and 23andMe cohorts. We report results for P+T, LDpred, P+T-funct-LASSO, AnnoPred, LDpred-funct-inf and LDpred-funct, for each of 4 training data sets: UK Biobank interim release (113,660 training samples), UK Biobank (408,092 training samples), 23andMe (698,430 training samples) and meta-analysis of UK Biobank and 23andMe (1,107,430 training samples). We also report results for a fixed-effect meta-analysis of UK Biobank and 23andMe. For SBayesR we obtained prediction R^2 of 0.0219, 0.3829, 0.2658, 0.3967 and 0.3028 respectively for the 5 data sets listed. For the UK Biobank interim release analysis, we obtained prediction R^2 of 0.0219 and $h_g^2 = 0.054$, perhaps because the algorithm failed to converge.

Method<br>Method	South Asians<br>Avg R^2	Africans<br>Avg R^2
P+T	0.0568 (0.0022)	0.0172 (0.0014)
LDpred	0.0666 (0.0031)	0.0241 (0.0018)
SBayesR	0.0724 (0.0032)	0.0250 (0.0019)
P+T-funct-LASSO	0.0631(0.0025)	0.0185 (0.0016)
AnnoPred	0.0744 (0.0032)	0.0282 (0.0036)
LDpred-funct-inf	0.0704 (0.0029)	0.0263 (0.0019)
LDpred-funct	0.0727 (0.0030)	0.0296 (0.0019)

Supplementary Table 28: Average prediction accuracy of 7 polygenic prediction methods in individuals of South Asian and African ancestry across 21 UK Biobank traits applied. We report the average prediction R^2 of each method in each population (block-jackknife s.e. in parentheses). We analyzed training samples of British ancestry from UK Biobank (average N=373K). We analyzed 7,444 unrelated South Asian validation samples from UK Biobank (74% Indian, 23% Pakistani, 3% Bangladeshi) and 7,379 unrelated African validation samples from UK Biobank (55% Caribbean, 45% African). In the South Asian validation sample, LDpred-funct attained +9.2%, +0.5%, -2.3%, +3.3% relative improvements in average prediction R^2 vs. LDpred, SBayesR, AnnoPred, LDpred-funct-inf respectively; $P < 5*10^{-5}$, $P = 0.8$, $P = 0.34$, $P < 7 * 10^{-5}$ for differences using two-sided z-test based on block-jackknife s.e. In the African validation sample, LDpred-funct attained +23%, +18%, +5.0%, +13% relative improvements in average prediction R^2 vs. LDpred, SBayesR, AnnoPred, LDpred-funct-inf respectively; $P < 10^{-5}$, $P < 2 * 10^{-3}$, $P = 0.2$, $P < 2 * 10^{-5}$ for differences using two-sided z-test based on block-jackknife s.e.

	Trait	h_g^2	LDpred-funct-inf under different priors: baselineLD (1000G)	baselineLD (UK10K)	baselineLD + LDAK (UK10K)
1	Height	0.575	0.4003	0.4022	0.4046
2	Hair color	0.446	0.2638	0.2778	0.2727
3	Platelet count	0.401	0.2290	0.2291	0.2303
4	Bone mineral density	0.398	0.2107	0.2109	0.2108
5	Balding type I	0.323	0.1078	0.1065	0.1082
6	Red blood cell count	0.319	0.1568	0.1571	0.1551
7	Age at menarche	0.313	0.1054	0.1052	0.1051
8	FEV1 FVC ratio	0.309	0.1289	0.1284	0.1286
9	Body mass index	0.307	0.1491	0.1498	0.1484
10	RBC distribution width	0.286	0.1394	0.1408	0.1404
11	Eosinophil count	0.274	0.1342	0.1350	0.1348
12	Forced vital capacity	0.274	0.1153	0.1150	0.1139
13	White blood cell count	0.273	0.1244	0.1254	0.1249
14	Systolic Blood pressure	0.267	0.1135	0.1144	0.1141
15	Tanning ability	0.235	0.1219	0.1227	0.1206
16	Waist hip ratio	0.210	0.0786	0.0786	0.0785
17	College Education	0.198	0.0770	0.0780	0.0777
18	Hyperthension	0.179	0.0527	0.0533	0.0536
19	Cardiovascular Diseases	0.160	0.0432	0.0434	0.0433
20	Morning Person	0.137	0.0413	0.0414	0.0415
21	Eczema	0.118	0.0279	0.0289	0.0282

Supplementary Table 29: Accuracy of LDpred-funct-inf(1000G), LDpred-funct-inf(UK10K) and LDpred-funct-inf(UK10K, baseline-LD+LDAK) across 21 UK Biobank traits. We report results for each trait. Results for Average across traits are reported in Supplementary Table 17.
