# Supplementary material for: Incorporating functional priors improves polygenic prediction accuracy in UK Biobank and 23andMe data sets
Source: Nat Commun. 2021 Oct 18;12:6052. doi: 10.1038/s41467-021-25171-9 (PMC8523709; doi:10.1038/s41467-021-25171-9)
Supplement: Supplementary file 3 — Reporting Summary [file 41467_2021_25171_MOESM3_ESM.pdf]

nature research

Corresponding author(s): C. Marquez-Luna; A.L. Price

Last updated by author(s): May 20, 2021

Reporting Summary

Nature Research wishes to improve the reproducibility of the work that we publish. This form provides structure for consistency and transparency in reporting. For further information on Nature Research policies, seeAuthors & Referees and theEditorial Policy Checklist .

Statistics

For all statistical analyses, confirm that the following items are present in the figure legend, table legend, main text, or Methods section.

n/a	Confirmed	
☐	☒	The exact sample size (*n*) for each experimental group/condition, given as a discrete number and unit of measurement
☐	☒	A statement on whether measurements were taken from distinct samples or whether the same sample was measured repeatedly
☐	☒	The statistical test(s) used AND whether they are one- or two-sided<br>*Only common tests should be described solely by name; describe more complex techniques in the Methods section.*
☐	☒	A description of all covariates tested
☐	☒	A description of any assumptions or corrections, such as tests of normality and adjustment for multiple comparisons
☐	☒	A full description of the statistical parameters including central tendency (e.g. means) or other basic estimates (e.g. regression coefficient) AND variation (e.g. standard deviation) or associated estimates of uncertainty (e.g. confidence intervals)
☐	☒	For null hypothesis testing, the test statistic (e.g. *F*, *t*, *r*) with confidence intervals, effect sizes, degrees of freedom and *P* value noted<br>*Give P values as exact values whenever suitable.*
☐	☒	For Bayesian analysis, information on the choice of priors and Markov chain Monte Carlo settings
☒	☐	For hierarchical and complex designs, identification of the appropriate level for tests and full reporting of outcomes
☐	☒	Estimates of effect sizes (e.g. Cohen's *d*, Pearson's *r*), indicating how they were calculated

Our web collection on statistics for biologists contains articles on many of the points above.

Software and code

Policy information about availability of computer code

Data collection	We analyzed raw genotype-phenotype data from UK Biobank (application 16549). Quality control analysis was primarily applied using Plink-2 and data files obtained from the UK Biobank described in Bycroft et al. 2018 Nature. Also, BOLT-LMM v2.3 and LDpred-funct apply quality control checks (details described in the Methods section).
Data analysis	Data analysis described in the Methods section was performed using the following softwares:<br>Software implementing the LDpred-funct-inf and LDpred-funct: https://www.hsph.harvard.edu/alkes-price/software (https://doi.org/10.5281/zenodo.4579879)<br>LDscore regression v1.0.1 software: https://github.com/bulik/ldsc<br>BOLT-LMM v2.3 software http://data.broadinstitute.org/alkesgroup/BOLT-LMM/<br>Software implementing LDpred: https://github.com/bvilhjal/ldpred<br>Software implementing Annopred: https://github.com/yiminghu/AnnoPred<br>SBayesR 2.0 software: http://cnsgenomics.com/software/gctb/<br>Plink 2.0 is available at: \url{https://www.cog-genomics.org/plink/2.0/}<br>FASTPCA is available in EIGENSOFT(7.2.1) at \url{https://github.com/DReichLab/EIG/archive/v7.2.1.tar.gz} (more details in {https://www.hsph.harvard.edu/alkes-price/software})<br>LDAK version 5 is available at \url{http://dougspeed.com/downloads/}

For manuscripts utilizing custom algorithms or software that are central to the research but not yet described in published literature, software must be made available to editors/reviewers. We strongly encourage code deposition in a community repository (e.g. GitHub). See the Nature Research guidelines for submitting code & software for further information.

Data

Policy information about availability of data

All manuscripts must include a data availability statement. This statement should provide the following information, where applicable:

- Accession codes, unique identifiers, or web links for publicly available datasets
- A list of figures that have associated raw data
- A description of any restrictions on data availability

Access to the UK Biobank resource is available via application in http://www.ukbiobank.ac.uk/
We used BOLT-LMM v2.3 association statistics available at: https://data.broadinstitute.org/alkesgroup/UKBB/UKBB_409K/
The baseline-LD annotations (v.2.1) used to compute functional enrichments in the primary analysis are available at https://alkesgroup.broadinstitute.org/LDSCORE/1000G_Phase3_baseline_v1.2_ldscores.tgz
1000 Genomes Project data is available at http://www.1000genomes.org/
Access to the UK10K data used in the secondary analysis is available via application at https://www.uk10k.org/data_access.html
23andMe height association statistics: The full summary statistics for the 23andMe height GWAS data will have restricted access and will be made available through 23andMe to qualified researchers under an agreement with 23andMe that protects the privacy of the 23andMe participants. Please visit https://research.23andme.com/collaborate/#publication for more information and to apply to access the data.
SBayesR shrunk, and sparse LD matrices can be downloaded from Zenodo public repository https://zenodo.org/, for both 1.09 million HapMap3 (10.5281/zenodo.3350914) and 2.8 million pruned variants (10.5281/zenodo.3375373).

Field-specific reporting

Please select the one below that is the best fit for your research. If you are not sure, read the appropriate sections before making your selection.

☒ Life sciences ☐ Behavioural & social sciences ☐ Ecological, evolutionary & environmental sciences

For a reference copy of the document with all sections, see nature.com/documents/nr-reporting-summary-flat.pdf

Life sciences study design

All studies must disclose on these points even when the disclosure is negative.

Sample size	We used the larger sample size that was available. For analysis of 21 highly heritable traits in the UK Biobank (average N=373K for training data and average 22K for validation data), we restricted the analysis to European-ancestry samples, independent UK Biobank traits with phenotyping rate >80%, and with SNP-heritability h2g > 0.2 for quantitative traits, observed-scale SNP-heritability h2g > 0.1 for binary traits. For height, a meta-analysis of UK Biobank and 23andMe cohorts led to 1107K samples for training data and 24K for validation.
Data exclusions	Our study was restricted to individuals of European ancestry within UK Biobank. We excluded variants based on quality control metrics, such as MAF, imputation quality, and excluded samples from the validation set related to training samples and/or other validation samples described in the Methods section of the manuscript.
Replication	We show that LDpred-funct attains higher polygenic prediction accuracy than other methods in simulations with real genotypes, analyses of 21 highly heritable UK Biobank traits, and meta-analyses of height using training data UK Biobank and 23andMe cohorts. We test our methods mainly in a European ancestry sample described in the Methods section and an African and South Asian population as described in the Discussion section. We believe the range of scenarios in simulations and real data analyses across multiple traits is evidence for reviewers to assess these conclusions.
Randomization	For genome-wide association studies involving the UK Biobank data, we used a linear-mixed-model (LMM) method implemented in BOLT-LMM v2.3. This approach is the best way to control confounders such as relatedness and population structure, and it is the standard way for this type of study. The 23andMe height summary statistics were computed using linear regression adjusting for age, gender, genotyping platform, and the top five principal components for accounting for residual population structure.
Blinding	Since we did not investigate any treatment, blinding in population data collection is not a concern for this study.

Reporting for specific materials, systems and methods

We require information from authors about some types of materials, experimental systems and methods used in many studies. Here, indicate whether each material, system or method listed is relevant to your study. If you are not sure if a list item applies to your research, read the appropriate section before selecting a response.

Materials & experimental systems

n/a	Involved in the study
☒	☐ Antibodies
☒	☐ Eukaryotic cell lines
☒	☐ Palaeontology
☒	☐ Animals and other organisms
☒	☐ Human research participants
☒	☐ Clinical data

Methods

n/a	Involved in the study
☒	☐ ChIP-seq
☒	☐ Flow cytometry
☒	☐ MRI-based neuroimaging
