# Supplementary material for: Incorporating functional priors improves polygenic prediction accuracy in UK Biobank and 23andMe data sets
Source: Nat Commun. 2021 Oct 18;12:6052. doi: 10.1038/s41467-021-25171-9 (PMC8523709; doi:10.1038/s41467-021-25171-9)
Supplement: Supplementary file 4 — Source Data [file 41467_2021_25171_MOESM4_ESM.zip › data source files/Description of Supplementary Files.docx]

**Description of Supplementary Files**

**File Name**: Supplementary Data 1.xlsx

**Title**: Supplementary Data 1. Accuracy of 6 polygenic prediction methods in simulations using UK Biobank genotypes.

**Description**: It includes raw data underlying Figure 1. We report results for P+T, LDpred, P+T-funct-LASSO, AnnoPred, LDpred-funct-inf and LDpred-funct in chromosome 1 simulations with 2,000 causal variants (sparse architecture) and 5,000 causal variants (polygenic architecture). Results are averaged across 100 simulations (column Average R2), differences vs. LDpred are represented ad bottom dashed lines in Figure 1. Top dashed line denotes simulated SNP-heritability of 0.5.

**File Name:** Supplementary Data 2.xlsx

**Title**: Supplementary Data 2. Accuracy of 7 polygenic prediction methods across 14 UK Biobank quantitative traits.

**Description**: It includes raw data underlying Figure 2. We report results for P+T, LDpred, SBayesR, P+T-funct-LASSO, AnnoPred, LDpred-funct-inf and LDpred-funct. Dashed lines denote estimates of SNP-heritability (H2g). We report average R2 obtained from 200 jackknife blocks, as well as the invidiual points for each jackknife block. Abbreviations: Red Blood Cell Distribution Width (RBD distribution width), forced expiratory volume in one second (FEV1) and forced vital capacity (FVC).

**File** **Name**: Supplementary data 3.xlsx

**Title**: Supplementary data 3. Accuracy of 7 polygenic prediction methods across 7 UK Biobank binary traits.

**Description**: It includes raw data underlying Figure 3. We report results for P+T, LDpred, SBayesR, P+T-funct-LASSO, AnnoPred, LDpred-funct-inf and LDpred-funct. Dashed lines denote estimates of SNP-heritability (H2g). We report average R2 obtained from 200 jackknife blocks, as well as the invidiual points for each jackknife block.

**File Name**: Supplementary data 4.xlsx

**Title**: Supplementary data 4. Accuracy of 6 prediction methods in height meta-analysis of UK Biobank and 23andMe cohorts.

**Description**: Data points from Figure 4.

**File Name:** Supplementary data 5.xlsx

**Title**: Accuracy of 6 polygenic prediction methods in simulations using UK Biobank genotypes, for 4 values of the number of causal variants.

**Description**: It includes raw data underlying Supplementary Figure 1. We report results for P+T, LDpred, P+T-funct-LASSO, AnnoPred, LDpred-funct-inf and LDpred-funct in chromosome 1 simulations with 1,000 causal variants (very sparse architecture), 2,000 causal variants (sparse architecture), 5,000 causal variants (polygenic architecture) and 10,000 causal variants (very polygenic architecture). Results are averaged across 100 simulations. Top dashed line denotes simulated SNP-heritability of 0.5. Bottom dashed lines denote differences vs. LDpred; error bars represent 95\% confidence intervals.

**File Name:** Supplementary data 6.xlsx

**Title:** Supplementary data 6. Relative improvement of LDpred-funct vs. LDpred as a function of h^2^_g_.

**Description:** It includes raw data underlying Supplementary Figure 2. We plot relative improvement vs. h^2^_g_ (measured on the observed scale for binary traits) for 19 UK Biobank traits; we excluded two sex-specific traits, age at menarche and balding type I.
